# Modulation of antiviral genes by African swine fever isolates of diverse virulence

**DOI:** 10.3389/fvets.2025.1591709

**Published:** 2025-06-03

**Authors:** Giulia Franzoni, Lorena Mura, Susanna Zinellu, Pedro J. Sanchez-Cordon, Miriam Pedrera, Silvia Dei Giudici

**Affiliations:** ^1^Department of Animal Health, Istituto Zooprofilattico Sperimentale della Sardegna, Sassari, Italy; ^2^Centro de Investigacion en Sanidad Animal (CISA), Instituto Nacional de Investigacion y Tecnologia Agraria y Alimentaria (INIA), Consejo Superior de Investigaciones Cientificas (CSIC), Valdeolmos, Madrid, Spain

**Keywords:** African swine fever virus, macrophages, interferon stimulatory genes, pattern recognition receptors, cytokines

## Abstract

African swine fever virus (ASFV), the aetiological agent of a devastating swine disease, has developed several strategies to replicate in porcine macrophages, its main target cells. In this work, we investigated the expression of 84 antiviral genes in macrophages infected with the virulent strain 26544/OG10 or the attenuated strain NH/P68. Infection with both strains caused an early activation of antiviral defenses, with up-regulation of RNA-sensing molecules and interferon-stimulating genes. However, as viral replication progresses, down-regulation of key inflammatory genes was observed, especially during infection with NH/P68, suggesting an impairment of macrophages' inflammatory response. Data generated provide a better portrait of ASFV immune evasion strategies.

## 1 Introduction

African swine fever virus (ASFV) is the etiologic agent of an infectious haemorrhagic disease of suids that poses a threat to the swine industry worldwide (1). Since 2007, ASF has spread rapidly around the world, with outbreaks in Africa, Europe, Asia, Oceania, and the Caribbean, with disease control hampered by the lack of available vaccines or treatments (2). There is urgent need to better understand ASFV immune evasion mechanisms to contribute to vaccine development (1).

ASF viruses have been group into 24 genotypes based on the partial sequence of the gene B646L (1). All the genotype are present in Africa, whereas in Europe and Asia mainly genotype I and II have been circulated in the last decades (1, 3). In addition, in recent years highly virulent ASFV strains containing genetic elements of both genotypes I and II ASF viruses (Recombinant ASFV Genotype I/II Strain) were detected in China and in Vietnam (4, 5).

ASFV isolates, regarless of the genotype, mainly target porcine macrophages and virulent isolates has developed several strategies to replicate efficiently in its target cells by impairing their defense mechanisms (6). These decoy strategies are partially lost in attenuated strains. ASFV strains of diverse virulence has been previously reported to differently modulate the expression of several cytokines by infected macrophages, including proinflammatory cytokines and type I interferons (IFNs) (7–9).

In the present study, we expanded the analysis on other key antiviral genes. Indeed, the innate immune response is the main line of defense against viral infections and involves several antiviral genes whose expression is induced by type I IFNs, specifically interferon-stimulated genes (ISGs).

The production of type I IFNs is induced by pathogen recognition receptors (PRRs), which recognize components from pathogens called pathogen-associated molecular patterns (PAMPs).

PRRs include NOD-like receptors (NLRs), toll-like receptors (TLRs) and RIG-I-like receptors (RLRs) (10). NLRs are cytoplasmic receptors that recognize PAMPs and may also act as key regulators of apoptosis and early development (11). On the other hand, some TLRs (TLR1, −2, -4, -5,−6) are expressed on the cell membrane and recognize microbial lipopeptides or lipopolysaccharides, whereas others are intracellular (TLR3,−7,−8,−9) and sense nucleic acids.

TLR activation results in initiation of intracellular signaling cascades leading to inflammasome activation (12). Inflammasome activation triggers the release of pro-inflammatory interleukins, TNF and chemokines; the consequent inflammatory responses counteract the progression of invading pathogens (13). Finally, RLRs are key sensors of viral infection, which mediate transcriptional induction of type I IFNs and other antiviral genes, and whose activation early in infection induces expression not only of type I IFNs, but also of pro-inflammatory cytokines (14). RLRs include DDX58 (also called RIG-I) and IFIH1 (also called MDA5), which are RNA sensors. For both receptors, viral recognition is facilitated by the DHX58 regulator (10).

In this study, we carried out a comparative evaluation of the ability of two strains of ASFV to modulate expression of antiviral genes in porcine macrophages. Two strains of ASFV genotype I with different *in vivo* pathogenicity were compared. On the one hand, the attenuated NH/P68, capable of causing only mild clinical signs in domestic pigs, such as fever and joint swelling (14). On the other hand, the highly virulent Sardinian strain 26544/OG10, capable of causing death in 10–14 days when administered to domestic pigs at very low doses (10 TCID_50_) using an intramuscular inoculation route (De Mia, unpublished results). The expression of key cytokines, PRRs, transcription factor, ISGs and other factors involved in antiviral defenses were evaluated.

## 2 Materials and methods

### 2.1 Animals

Four clinically healthy crossbred pigs (*Sus scrofa domesticus*), 6 to 18 months of age, were used as blood donors. Animals were housed at the Experiment Station of Istituto Zooprofilattico Sperimentale (IZS) of Sardinia, and were screened for ASFV, porcine circovirus 2 (PCV2), porcine parvovirus (PPV), porcine reproductive and respiratory syndrome virus (PRRSV), and *Mycoplasma hyopneumoniae*, to confirm their negative status, as previously described (15). Animal handling and experimental procedures (bleeding) were authorized by the Ministry of Health (Ref. 1232/2020-PR).

### 2.2 Viruses

Working stocks of the virulent Sardinian 26544/OG10 strain (GenBank accession number KM102979) and the low virulence NH/P68 strain (GenBank accession number NC044943) were obtained by virus propagation *in vitro* on sub-confluent monolayers of 2-day-old monocytes/macrophage cultures, following previous protocols (16). Mock virus supernatants were prepared in an identical manner without viral addition. Viral titers were determined using previously described protocols, calculated using the Spearman–Kärber formula and expressed as TCID_50_/mL (7).

### 2.3 Generation of monocyte-derived macrophages and infection

Monocyte-derived macrophages (moMΦ) were obtained *in vitro* by cultivating blood monocytes in media supplemented with recombinant human M-CSF (hM-CSF) (Thermo Fisher Scientific, Waltham, MA, USA), as previously described (7). After 7 days in culture, moMΦ were detached, counted and seeded in 12-well plates. 24 h post-seeding, culture supernatant was removed and cells were infected with virulent strain 26544/OG10 or attenuated strain NH/P68, using a multiplicity of infection (MOI) of 1. Mock-infected controls were included in all the experiments.

Cells were cultured at 37°C 5% CO_2_. Virus inoculum was removed after 90 min of incubation, then moMΦ were washed with RPMI-1640 medium, and fresh RPMI-1640 supplemented with 10% FBS and antibiotics (cRPMI) was added to the wells (1.5 mL/well).

### 2.4 Gene expression analysis and flow cytometry

Cells were collected at 3 and 21 h post infection (hpi) for RNA extraction. RNA was extracted using a miRNAeasy Mini Kit (QIAGEN), then genomic DNA was digested using an RNase-Free DNase set (QIAGEN), and the concentration of RNA was determined using a Qubit 4 fluorometer (Thermo Fisher). Total RNA (500 ng) was used for cDNA synthesis using a RT2 First Strand Kit (QIAGEN). Real-time PCR was then conducted using an RT2 Profiler PCR Array for pig antiviral genes (QIAGEN, Cat. No. PASS-122Z). Data were normalized using the housekeeping genes present in the array (ACTB, B2M, GAPDH, HPRT1, and RPLP0). The relative gene expression levels compared to the mock-infected control were then calculated using the ΔCt method (2^−ΔΔCt^) (17). In parallel, intracellular levels of ASFV viral proteins (early p30 and late p72) were monitored at 21 hpi by flow cytometry, using a FACS Celesta (BD Biosciences), as previously described (7).

### 2.5 Statistical analysis

Flow cytometric data were analyzed using a BD FACS Diva Software, as previously described (7), whereas PCR array for 84 antiviral genes were analyzed using the Gene Globe Data Analysis Center available at QIAGEN (https://geneglobe.qiagen.com/us/analyze, accessed on 14 February 2025). Both flow cytometry assays and PCR arrays were measured on macrophage subsets generated using four blood donor pigs (four biological replicates). For each animal, three conditions were obtained: mock-infected NHV-infected, 26544/OG10-infected. For each ASFV strain, fold change in gene expression relative to the mock-infected control was calculated. The p values were calculated based on a Student's t-test of the replicate 2^(−ΔCt)^ values for each gene in the mock-infected and ASFV-infected groups; statistically significant difference was set as *p* < 0.05. GraphPad Prism 10.01 (GraphPad Software Inc., La Jolla, CA, USA) was used to graphically analyze the data. The PCR array data were presented as a heat map.

## 3 Results

**Figure 1:**
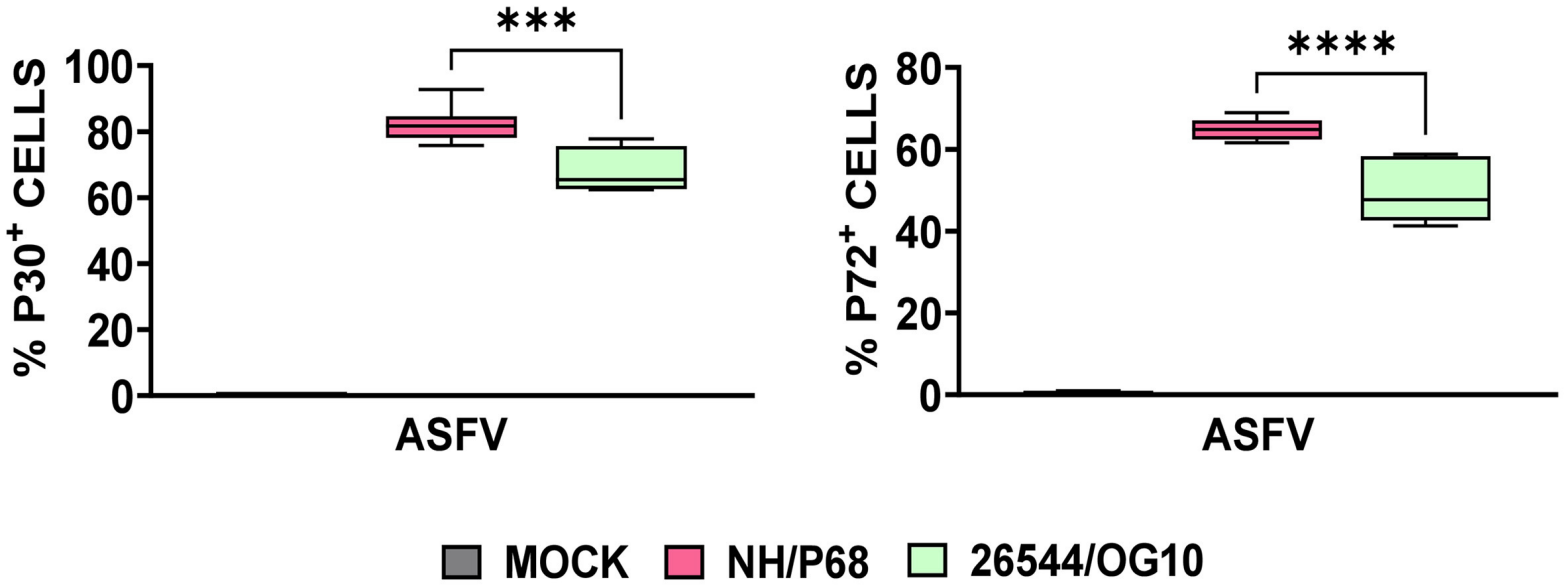
Intracellular levels of early and late viral protein in ASFV-infected moMΦ 21 h post-infection (hpi). Porcine moMΦ were infected with the attenuated NH/P68 or the virulent 26544/OG10, using a MOI of 1, alongside mock-infected controls. 21 hpi, intracellular levels of the early protein p30 and the late protein p72 of ASFV were analyzed by flow cytometry. Data from four independent experiments utilizing different blood donors are presented. NH/P68 and 26544/OG10 data were compared using an unpaired *t*-test; ^***^p < 0.001, ^****^p < 0.0001.

**Figure 2:**
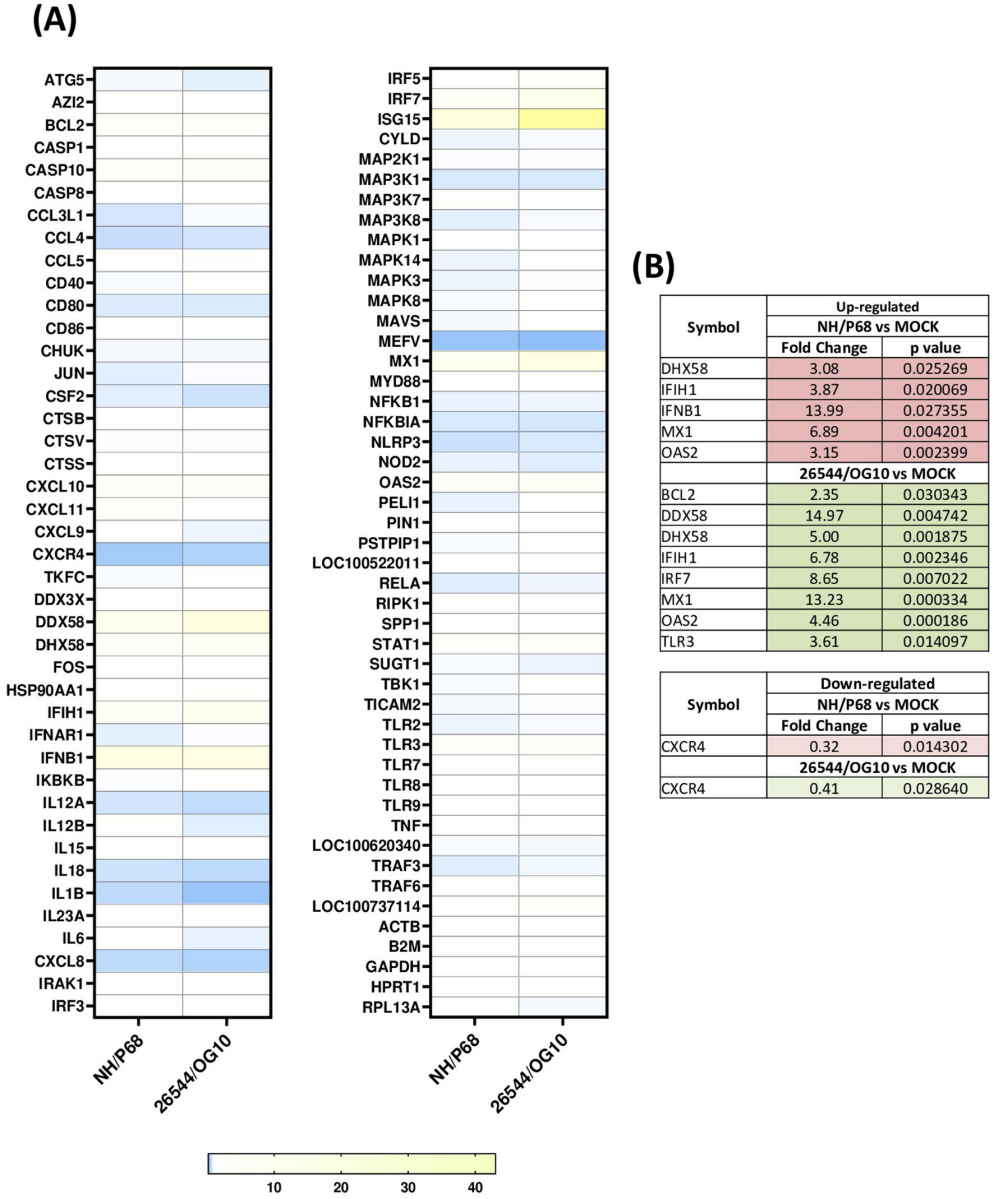
Modulation of 84 antiviral genes in moMΦ 3 h post-infection (hpi). Porcine moMΦ were infected with the attenuated NH/P68 or the virulent 26544/OG10, using a MOI of 1, alongside mock-infected controls. 3 hpi, macrophages were analyzed using the RT2 Profiler PCR Array for 84 antiviral genes. **(A)** The heatmap illustrates fold change expression of immune-related genes. Data from four independent experiments utilizing different blood donors are presented. For each ASFV strain, fold change in gene expression relative to the mock-infected control was calculated, and colors represent these fold changes: yellow represents the highest value, white the baseline value (fold change = 1), and blue the smallest value. **(B)** Statistically significantly up-regulated (fold change > 2, *p*-value < 0.05) and down-regulated (fold change < 0.5, *p-*value < 0.05) genes are presented, with the corresponding fold change and *p-*value.

**Figure 3:**
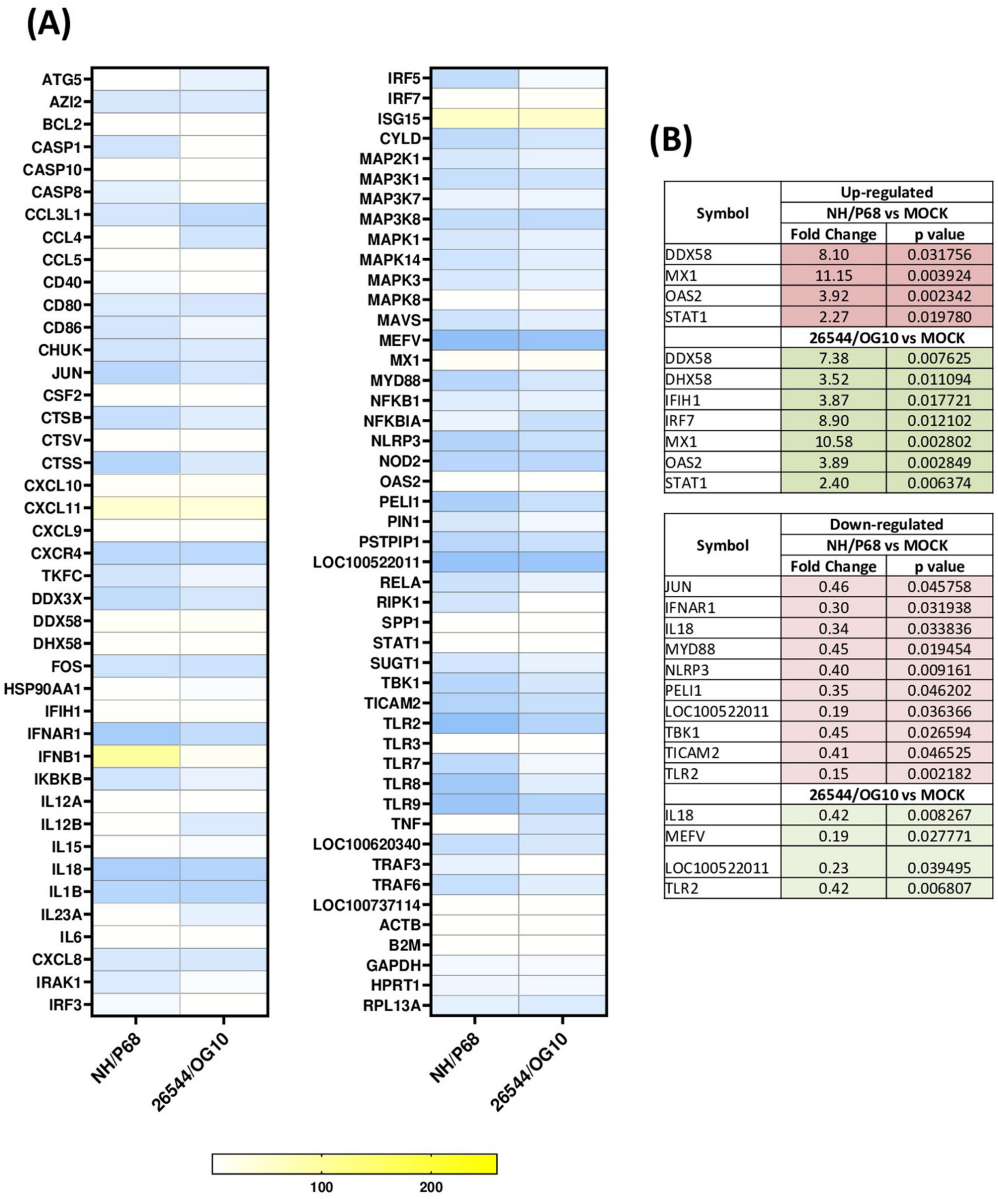
Modulation of 84 antiviral genes in moMΦ 21 h post-infection (hpi). Porcine moMΦ were infected with the attenuated NH/P68 or the virulent 26544/OG10, using a MOI of 1, alongside mock-infected controls. 21 hpi, macrophages were analyzed using the RT2 Profiler PCR Array for 84 antiviral genes. **(A)** The heatmap illustrates fold change expression of immune-related genes. Data from four independent experiments utilizing different blood donors are presented. For each ASFV strain, fold change in gene expression relative to the mock-infected control was calculated, and colors represent these fold changes: yellow represents the highest value, white the baseline value (fold change = 1), and blue the smallest value. **(B)** Statistically significantly up-regulated (fold change > 2, *p*-value < 0.05) and down-regulated (fold change < 0.5, *p*-value < 0.05) genes are presented, with the corresponding fold change and *p*-value.

Using this experimental setup (MOI = 1), we observed that almost 80% of NH/P68-infected moMΦ and 60–70% of 26544/OG10-infected moMΦ presented early p30 protein of ASFV at 21 hpi (Figure 1). At the same time point, almost 60% of NH/P68-infected moMΦ and 40–50% of 26544/OG10-infected moMΦ expressed intracellular levels of late p72 protein of ASFV (Figure 1), in line with previously published studies (7). In Figure 2, expression levels of 84 key antiviral genes at 3 hpi (early time pi) are presented, whereas array results at 21 hpi (late time pi) are presented in Figure 3. In both figures, panel B shows a list of up-regulated (fold-change > 2) and down-regulated (fold change < 0.5) genes with statistical significance (*p* < 0.05).

At 3 hpi, our data revealed that infection with both isolates produced an up-regulation of genes coding for molecules with strong antiviral activity such as *IFNB1* (statistically significant for NH/P68, *p* = 0.027355) and three ISGs (*MX1, OAS2, and ISG15*), although the latter without statistical significance. Infection with both isolates resulted also in up-regulation of genes coding for two receptors sensing RNA (*DDX58* and *IFIH1*), as well as the related regulator *DHX58*. For *DDX58*, statistical significance was observed only for 26544/OG10 (*p* = 0.004742) and a tendency was detected in infection with NH/P68 (*p* = 0.081272). At the same time point, 26544/OG10 infection resulted in increased *TLR3* receptor, *IRF7* transcription factor, and *BCL2* expression (Figure 2). BCL2 is indeed a regulator of apoptosis and its early up-regulation should contribute to prevent apoptosis of infected cells (6). At 3 hpi, only few genes were down-regulated with fold-change < 0.5, including for both viruses genes encoding for chemokine receptor *CXCR4* and for pyrin (*MEFV*), an important modulator of inflammation, although only *CXCR4* with statistical significance (Figure 2).

At 21 hpi, our data revealed that infection with both isolates resulted in up-regulation of several key immune genes, some of which also expressed at 3 hpi. Prominent among these were *IFNB1* and three ISGs (*MX1, OAS2, and ISG15*), the latter with a high fold-change (>50), but without statistical significance. Also at this time point, both isolates triggered up-regulation of genes coding for two receptors sensing RNA (*DDX58* and *IFIH1*), as well as the regulator *DHX58*, although for *IFIH1* and *DHX58* statistical significance was only observed for 26544/OG10 (Figure 3). At 21 hpi, increased expression of the transcription factors *STAT1* and *IRF7* was also observed, the latter with significance for 26544/OG10 (*p* = 0.012102) and a tendency for NH/P68 (*p* = 0.099827) (Figure 3). Differences between strains were observed. Thus, the expression levels of *DDX58, DHX58, IFIH1, MX1, and OAS2* decreased slightly as infection with 26544/OG10 progressed (higher fold-changes at 3 hpi compared to 21 hpi). In contrast, in the case of attenuated NH/P68, the fold-changes values of *MX1, OAS2* increased slightly as infection progressed (Figures 2, 3). At 21 hpi, for *IFNB1* we observed a fold-change > 261 for NH/P68, more than ten-fold higher than observed for 153 26544/OG10 (fold-change = 14.07), although without statistical significance (Figure 3). At 21 hpi several genes were down-regulated with fold-change < 0.5, especially in infection with NH/P68. Both strains significantly down regulated the genes encoding for the receptor *TLR2* as well as the pro-inflammatory cytokine *IL18* and *LOC100522011*. Infection with these viruses also resulted in down-regulation of *MEFV*, with statistical significance for 26544/OG10 (*p* = 0.027771) and a tendency for NH/P68 (*p* = 0.079195). Infection with NH/P68 also caused down-regulation of *IFNAR1* and *NLRP3* receptors, *MYD88* adaptor proteins, as well as other molecules involved in modulating receptor signaling pathways (*JUN, PELI1, TBK1, and TICAM2*) (Figure 3). For NH/P68, a tendency was also observed for other intracellular receptors, such as *NOD2* (*p* = 0.085491), *TLR8* (*p* = 0.079816), and *TLR9* (*p* = 0.099827), whereas for 26544/OG10 a tendency was observed only for *NOD2* (*p* = 0.08878) (Figure 3).

## 4 Discussion

In this study, we analyzed the impact of ASFV infection on antiviral genes expression in porcine macrophages. Two strains with diverse virulence were compared: the highly virulent 26544/OG10 and the attenuated NH/P68, the latter able to induce good levels of protection to challenge with virulent isolates belonging to either genotype I and II isolates (14, 18). Expression of antiviral genes were evaluated at early stage post-infection (3 hpi) and at later time point (21 hpi). In line with our previous study, at 21 hpi almost 50% of porcine moMΦ infected with 26544/OG10 stained positive for the late ASFV viral protein p72, but the attenuated NH/P68 infected porcine moMΦ more efficiently than the virulent strain 26544/OG10 (7).

Our data revealed that infection with both strains resulted in high expression of several antiviral genes, including *IFNB*, some ISGs (*MX1, OAS2*, and *ISG15*), RIG-I receptors (*DDX58* and *IFIH1*), as well as the regulator *DHX58* and the transcription factor *IRF-7*, which corroborated an activation of the innate immune defense as early as 3 hpi. Our results are consistent with previous studies (9, 19), in which transcriptome analysis showed that infection of porcine alveolar macrophages with the virulent genotype II strain HLJ/18 (MOI = 1) or the attenuated HuB20 (MOI = 3) resulted in increased expression of cytokine-related genes and ISGs, including *MX1*, as well as the transcription factor *STAT1* (9, 19). Nevertheless, in the same study researchers observed that expression of several antiviral genes correlating negatively with ASFV viral loads. In this sense, the expression levels of *STAT1*, which is a key transcription regulator of the type I IFN signaling, decreased alongside viral replication, suggesting that despite an initial activation of host defenses, increased replication of the HLJ/18 strain progressively suppressed cellular resistances (19). Similar results were observed with the virulent strain used in our study (26544/OG10), where expression levels for some key antiviral genes (*DDX58, DHX58, IFIH1, MX1, and OAS2*) decreased slightly as infection progressed. This was not observed for the attenuated NH/P68, where progression of the infection resulted in increased expression of *MX1, OAS2, IFNB1*. Regarding type I IFN, we and others had previously reported that ASFV triggered early up-regulation of *IFNB* (8, 20–22), albeit with differences between strains of different virulence. Thus, infection with the virulent genotype I Benin or genotype II Armenia/07 strains resulted in a mild increase in *IFNB* mRNA levels, whereas infection with the low virulence genotype I OURT88/3 or NH/P68 strain triggered a sustained up-regulation of this gene (21, 22). In agreement, in our study we observed a prominent increase of *IFNB* expression as infection with NH/P68 progressed (21 hpi), with a fold-change > 261, more than ten fold higher than that observed in infection with virulent strain 26544/OG10 at the same time-point. IFN-β is indeed a key mediator between the innate and adaptive immune response, thus its enhanced expression should translate into better control of virus dissemination in the host (6).

In parallel, we detected down-regulation of key inflammatory genes after infection with both NH/P68 and 26544/OG10. At 3 hpi, only few genes were down-regulated with a fold-change < 0.5, and among them only *CXCR4* with statistical significance. This receptor is specific for the chemokine *CXCL12*, which possesses potent chemotactic activity for lymphocytes (23). Early down-regulation of this gene could hinder the recruitment of innate immune cells, which could delay the development of a protective immune response. In contrast to what occurred at 3 hpi, down-regulation of a greater number of genes was observed at 21 hpi. Both strains down-regulated the expression of *TLR2*, the pro-inflammatory cytokine *IL18*, and *MEFV* (significant only for 26544/OG10), the latter a gene encoding for pyrin, involved in the assembly of inflammasome complexes (24). Overall, down-regulation of these genes decreases host inflammatory and anti-microbial responses, facilitating the progression of invasive pathogens, such as ASFV. These data would be in line with previous studies in which infection of moMΦ with the virulent genotype II strain Georgia 2007 (MOI = 1) triggered a decrease in the expression of several TLRs, notably among these *TLR2* and *TLR4* (20). This study suggested that this fact compromised the ability of ASFV-infected macrophage to polarize toward a pro-inflammatory and anti-microbial phenotype (20). In the same study, infection of moMΦ with Georgia 2007 strain down-regulated other receptors, such as interferon receptors (*IFNAR1, IFNAR2, IFNGR*) and interleukin receptors (*IL4R, IL10RA, IL10RB*, and *IL17RA*), as well as transcriptomic factors (e.g., *FOS, JUN, and TBK1*) (20). Transcriptome analysis of ASFV-infected alveolar macrophages, in which the virulent genotype II strain HLJ/18 was used, also revealed strong inhibition of host immunity-related genes, including *TLR2, TLR3*, and *TLR8* (25). Other studies, carried out with genotype I strains of different virulence (Benin 97/1, NH/P68, 22653/14) reported that ASFV down-regulated CD14 and CD16 expression in infected cells (7), likely impairing macrophage's anti-microbial activity (6). Therefore, studies on genes down-regulation following infection with high and low virulence ASFV strains suggest an impairment of macrophage anti-viral activity, which might facilitate virus replication within these cells, with recruitment of elements of the macrophage translation machinery to viral factories and production of new infectious particles (6).

Of note, infection with the attenuated NH/P68 strain also resulted in a more marked down-regulation of antimicrobial genes at 21 hpi compared to virulent strain 26544/OG10. Specifically, *JUN, IFNAR1, MYD88, NLRP3, PELI1, TBK1, and TICAM2* were statistically significantly down-regulated. As in the present study, it has previously been shown that NH/P68 presented a more pronounced inhibitory effect compared to other virulent genotype I strains (such as the Sardinian 22653/14), a fact that might be related to the higher replication efficiency of NH/P68 early after infection compared to strains of higher virulence (7). In the context of attenuated strain infections, down-regulation of certain genes involved in the inflammatory response could also be related to the control of inflammatory signaling pathways in infected macrophages, which would control the cytokine storm and minimize the tissue damage, features that, however, characterize infections by highly virulent strains “*in vivo.”*

Taken together, our data highlighted that infection with both the attenuated strain NH/P68 and the virulent strain 26544/OG10 resulted in early activation of innate immune defenses. Nevertheless, expression of some of these antiviral genes decreased as replication of the virulent 26544/OG10 progressed, but that was not observed for the attenuated NH/P68. In parallel, infection with either strain resulted in down-regulation of several antiviral genes, suggesting that infection progressively impair macrophage's defenses allowing viral replication. At 21 hpi, infection with the attenuated NH/P68 strain triggered a more marked down-regulation of pro-inflammatory genes compared to the virulent 26544/OG10 strain, as well as a more sustained increased in *IFNB* expression. That might contribute to better infection control and a non-exacerbated inflammatory response, avoiding the development of the “cytokine storm” and related tissue damage often observed during acute infection with virulent isolates. The data generated in the present study will provide a better portrait and understanding of ASFV immune evasion strategies, which is expected to contribute in the rational design of safe and efficient ASFV vaccines.

## Data Availability

Publicly available datasets were analyzed in this study. This data can be found here: https://www.ncbi.nlm.nih.gov/ with accession numbers (KM102979) and (NC044943).

## References

[1] Blome S Franzke K Beer M. African swine fever–a review of current knowledge. Virus Res. (2020) 287:198099. 10.1016/j.virusres.2020.198099 32755631

[2] OIE WAHIS Interface. Available online at: https://wahis.oie.int/#/dashboards/country-or-disease-dashboard (accessed 19 May, 2025).

[3] Sun E Huang L Zhang X Zhang J Shen D Zhang Z. Genotype I African swine fever viruses emerged in domestic pigs in China and caused chronic infection. Emerg Microbes Infect. (2021) 10:2183–93. 10.1080/22221751.2021.1999779 34709128 PMC8635679

[4] Zhao D Sun E Huang L Ding L Zhu Y Zhang J. Highly lethal genotype I and II recombinant African swine fever viruses detected in pigs. Nat Commun. (2023) 14:3096. 10.1038/s41467-023-38868-w 37248233 PMC10226439

[5] Le VP Nguyen VT Le TB Mai NTA Nguyen VD Than TT. Detection of recombinant African Swine fever virus strains of p72 Genotypes I and II in domestic Pigs, Vietnam, 2023. Emerg Infect Dis. (2024) 30:991–4. 10.3201/eid3005.231775 38666642 PMC11060461

[6] Wu L Yang B Yuan X Hong J Peng M Chen JL. Regulation and evasion of host immune response by African swine fever virus. Front. Microbiol. (2021) 12:698001. 10.3389/fmicb.2021.698001 34566910 PMC8457549

[7] Franzoni G Razzuoli E Dei Giudici S Carta T Galleri G Zinellu S. Comparison of macrophage responses to african swine fever viruses reveals that the NH/P68 strain is associated with enhanced sensitivity to type I IFN and cytokine responses from classically activated macrophages. Pathogens. (2020) 9:209. 10.3390/pathogens9030209 32178332 PMC7157553

[8] Razzuoli E Franzoni G Carta T Zinellu S Amadori M Modesto P. Modulation of type I interferon system by african swine fever virus. Pathogens. (2020) 9:361. 10.3390/pathogens9050361 32397378 PMC7281450

[9] Lv L Zhang T Jia H Zhang Y Ahsan A Zhao X. Temporally integrated transcriptome analysis reveals ASFV pathology and host response dynamics. Front Immunol. (2022) 13:995998. 10.3389/fimmu.2022.995998 36544767 PMC9761332

[10] Bourdon M Manet C Montagutelli X. Host genetic susceptibility to viral infections: the role of type I interferon induction. Genes Immun. (2020) 21:365–79. 10.1038/s41435-020-00116-2 33219336 PMC7677911

[11] Kim YK Shin JS Nahm MH. NOD-Like Receptors in Infection, Immunity, and DiseasesYonsei. Med J. (2016) 57:5–14. 10.3349/ymj.2016.57.1.5 26632377 PMC4696971

[12] Franchi L Munoz-Planillo R Nunez G. Sensing and reacting to microbes through the inflammasomes. Nat Immunol. (2012) 13:325–32. 10.1038/ni.2231 22430785 PMC3449002

[13] Rehwinkel J Gack MU. RIG-I-like receptors: their regulation and roles in RNA sensing. Nat Rev Immunol. (2020) 20:537–51. 10.1038/s41577-020-0288-3 32203325 PMC7094958

[14] Leitao A Cartaxeiro C Coelho R Cruz B Parkhouse R Portugal FC. The non-haemadsorbing African swine fever virus isolate ASFV/NH/P68 provides a model for defining the protective anti-virus immune 330 response. J Gen Virol 82. (2001) 82:513–23. 10.1099/0022-1317-82-3-513 11172092

[15] Dei Giudici S Franzoni G Bonelli P Angioi PP Zinellu S Deriu V. Genetic characterization of porcine circovirus 3 strains circulating in sardinian pigs and wild boars *Pathogens*. (2020) 9:344. 10.3390/pathogens9050344 32370251 PMC7280999

[16] OIE—World Organisation for Animal Health (Ed.) *Manual of Diagnostic Tests and Vaccines for Terrestrial Animals, 8th ed*. Paris, France: OIE. (2018)

[17] Livak KJ Schmittgen TD. Analysis of relative gene expression data using real-time quantitative PCR and the 2(-DeltaDeltaC(T)) method. Methods. (2001) 25:402–8. 10.1006/meth.2001.1262 11846609

[18] Gallardo C Sánchez EG Pérez-Núñez D Nogal M de León P Carrascosa ÁL. African swine fever virus (ASFV) protection mediated by NH/P68 and NH/P68 recombinant live-attenuated viruses. Vaccine. (2018) 36:2694–704. 10.1016/j.vaccine.2018.03.040 29609966

[19] Zheng Y Li S Li SH Yu S Wang Q Zhang K. Transcriptome profiling in swine macrophages infected with African swine fever virus at single-cell resolution. Proc Natl Acad Sci U.S.A. 119 (2022) 119:e2201288119. 10.1073/pnas.2201288119 35507870 PMC9171760

[20] Zhu JJ Ramanathan P Bishop EA O'Donnell V Gladue DP Borca MV. Mechanisms of African swine fever virus pathogenesis and immune evasion inferred from gene expression changes in infected swine macrophages. PLoS One. (2019) 14:e0223955. 10.1371/journal.pone.0223955 31725732 PMC6855437

[21] García-Belmonte R Pérez-Núñez D Pittau M Richt JA Revilla Y. African Swine Fever Virus Armenia/07 Virulent Strain Controls Interferon Beta Production through the cGAS342STING Pathway. J Virol. (2019) 93:e02298–18. 10.1128/JVI.02298-18 30918080 PMC6613762

[22] Reis AL Abrams CC Goatley LC Netherton C Chapman DG Sanchez-Cordon P. Deletion of African swine fever virus interferon inhibitors from the genome of a virulent isolate reduces virulence in domestic pigs and induces a protective response. Vaccine. (2016) 34:4698–4705. 10.1016/j.vaccine.2016.08.011 27521231 PMC5012891

[23] Busillo JM Benovic JL. Regulation of CXCR4 signaling. Biochim Biophys Acta. (2007) 1768:952–63. 10.1016/j.bbamem.2006.11.002 17169327 PMC1952230

[24] Heilig R Broz P. Function and mechanism of the pyrin inflammasome. Eur J Immunol. (2018) 48:230–238. 10.1002/eji.201746947 29148036

[25] Ju X Li F Li J Wu C Xiang G Zhao X. Genome-wide transcriptomic analysis of highly virulent African swine fever virus infection reveals complex and unique virus host interaction. Vet Microbiol. (2021) 261:109211. 10.1016/j.vetmic.2021.109211 34481273

